# General Treatment and Ophthalmic Management of Peters’ Anomaly

**DOI:** 10.3390/jcm13020532

**Published:** 2024-01-17

**Authors:** Bogumil Wowra, Dariusz Dobrowolski, Mohit Parekh, Edward Wylęgała

**Affiliations:** 1 Chair and Department of Ophthalmology, Faculty of Medical Sciences, Zabrze Medical University of Silesia, 40-760 Katowice, Poland; 2 Department of Ophthalmology, Saint Barbara Hospital, Trauma Center, 41-200 Sosnowiec, Poland; 3 Institute of Ophthalmology, University College London, London EC1V 9EL, UK

**Keywords:** Peters’ anomaly, keratoplasty, clinical findings, treatment, management, keratoprosthesis

## Abstract

Peters’ anomaly (PA) is a manifestation of complex disorders in the development of the anterior segment of the eye. The most recognizable feature of the disease is a doughnut-shaped central corneal opacity and adhesions between the opacity and underlying iris. Glaucoma is observed in 30–70% of patients, with up to 50% of the patients showing concomitant vision-threatening disorders. Up to 60% of patients have systemic abnormalities or developmental delays. Being a rare malformation, PA is one of the most common congenital indications for corneal transplantation in infants. Penetrating keratoplasty is used as the primary method of treatment in cases with corneal opacification of a degree that forbids visual development in both eyes. The heterogeneity of co-occurring ophthalmic and systemic malformations in the spectrum of PA determines the wide range of success, defined by various endpoints: graft clarity or visual acuity. Although surgical advancement has made corneal grafting possible in younger children, it has a higher graft failure rate and worse visual prognosis than adult keratoplasty. Optical sector iridectomy, pupil dilation, or cornea rotation can alternatively be performed. Satisfying results of pediatric keratoprosthesis in particular cases of PA have been described. Postoperative treatment of PA aims to maintain a clear optical pathway and prevent amblyopia. This article therefore aims at reporting the ophthalmic treatment and need for multidisciplinary management of PA, including pharmacological and surgical treatment.

## 1. Introduction

Peters’ anomaly (PA), a subtype of anterior segment dysgenesis, causes corneal opacity during development, with 44–60 annual cases reported in the United States. It involves iris, corneal endothelium, and Descemet’s membrane abnormalities, categorized as Peters type I or II (bilateral with lens abnormalities). Bilateral cases show a stronger association (71.8%) with systemic malformations than unilateral cases (36.8%). Peters plus syndrome, a variant, exhibits short stature, developmental delays, dysmorphic facial features, and systemic malformations in 60% of patients. Ocular complications include glaucoma, sclerocornea, corectopia, iris hypoplasia, cataract, ICE syndrome, aniridia, iris coloboma, persistent fetal vasculature, and microcornea, impacting infant visual development [1,2].

Significant progress has been achieved in understanding the molecular and developmental causes of congenital anomalies within the anterior segment of the eye, i.e., structures lying between the front surface of the cornea and the front surface of the vitreous. PA, a disorder representing a wide spectrum of anterior segment dysgenesis (ASD), is defined as a central congenital corneal opacity (CCO) associated with a posterior defect in Descemet’s membrane and the endothelium [3,4]. Its nomenclature is derived from the original findings published by Dr. Peters in 1906 [5,6]. CCO is diagnosed in 2,2–3,1 out of 100,000 births [1,7]. Despite being a rare congenital malformation, PA is the most common cause of CCO (40.3–65% of the cases) [8,9,10] and indication for corneal transplantation in infants in the United States [8,11]. Due to it being classified as a rare genetic disorder, the literature on PA is limited. This article therefore endeavors to report on the ophthalmic treatment of PA and emphasize the imperative for multidisciplinary management, encompassing both pharmacological and surgical interventions. 

## 2. Etiology

A variety of genes involved in the development of the anterior segment of the eye contribute to the large range and diversity of ASD. Some cases include a broad spectrum of abnormalities and are difficult to classify. It is therefore essential to understand PA as a consequence of disorders between the fourth and seventh gestation week. A defect in the homeotic genes causes the abnormal migration and differentiation of neural crest cells in the anterior segment, resulting in faulty separation of the lens from the surface ectoderm or aberrant reattachment of the lens/iris to the cornea [3,4,12]. It has been found that the majority of PA cases lack an appropriate genetic diagnosis. Reported mutations in the genes contributing to PA include COL4A1, CYP1B1, FLNA, FOXC1, FOXE3, HCCS, NDP, PAX6, PITX2, PITX3, SLC4A11, and TFAP2 [13,14]. The majority of cases are sporadic; however, both dominant and recessive inheritance patterns have been found [3,4,15].

## 3. Clinical Findings

**Figure 1:**
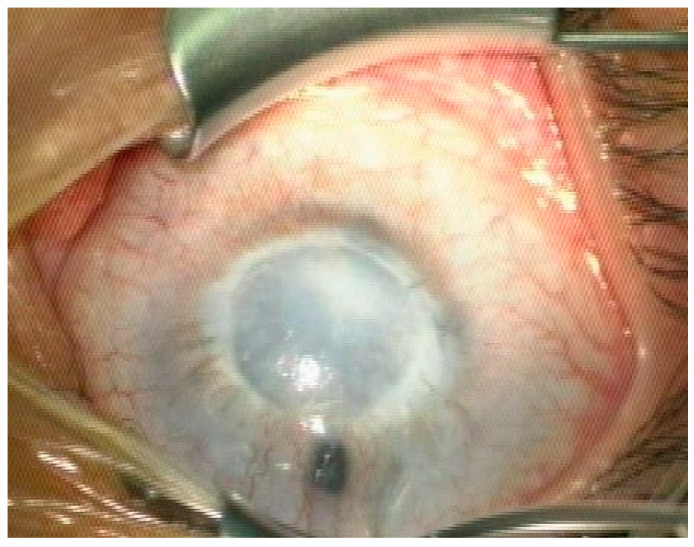
PA and congenital cataract 7 years after PK with subsequent partial lensectomy, qualified for re-transplantation due to opaque corneal graft, secondary retrocorneal membrane, and residual cataract.

PA is commonly classified into two types. Type 1 shows central or paracentral corneal opacity with iridocorneal adhesions, often unilateral, identified as “mild”; and type 2 presents with lens abnormalities (corneolenticular adhesions, cataract (Figure 1), degenerated lens within adherent anterior and posterior lens capsule), often bilateral, identified as “severe” [16]. 

**Figure 2:**
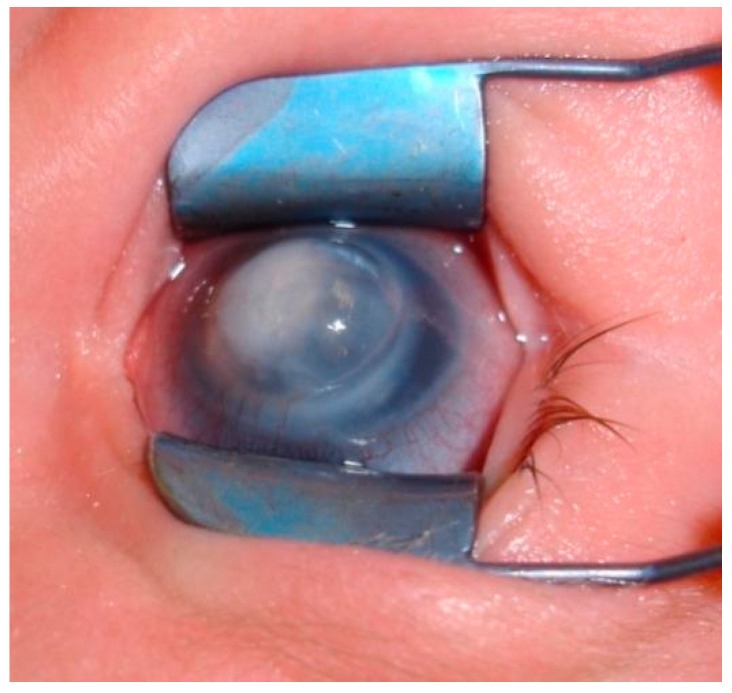
Peters’ anomaly and glaucoma.

**Figure 3:**
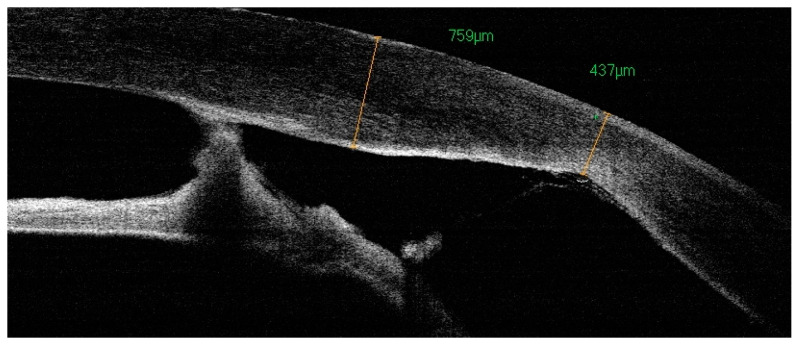
OCT: irregular corneal thickness and corresponding iris-endothelial adhesions.

Additionally, Peters plus syndrome is usually observed with the coexistence of various systemic anomalies. There are recommendations for modern nomenclature based on genetics and histopathology which classify PA into a group of congenital corneal opacities secondary to the maldevelopment of the anterior segment (kerato-irido-lenticular dysgenesis) [12]. Its progression initiates with a defect in the central corneal endothelium and Descemet’s membrane with corneal edema. The edema spreads beyond the defect, resulting in central leukoma. A doughnut-shaped congenital opacification is highly suspicious of PA. Typically, irido-corneal adhesions and synechiae are found in the periphery of corneal leukoma. Although the peripheral cornea is usually relatively clear, varying degrees of clouding may be present [10]. Fifty percent of cases of PA are associated with ocular abnormalities. The findings are diverse, but they all are sequelae of the retina at a particular stage of ocular embryology [12,17]. Although it may not be present at the outset, PA patients are at risk of developing glaucoma [10], which has been observed in 30–70% of the cases [10,17,18]. PA is found to be bilateral in 25–80% cases [10,19,20], whereas 21.3–60% of patients have systemic abnormalities or developmental delays [4,10,18,19]. According to Bhandari et al. there is a stronger association of bilateral than unilateral PA with systemic malformations (71.8% vs. 36.8%) [3], while Chang et al. showed no significant association between laterality and systemic anomalies [19]. PA accompanied by other ocular anomalies is more often connected with systemic anomalies [18]. However, the location or severity of corneal opacity have not been found to be correlated with the presence of systemic anomalies [18]. The majority of systemic abnormalities have their origin in primary non-ocular neural crest cells, which form the cartilage, bone, connective tissue, teeth components (except enamel), pigment cells, and peripheral nervous system of the face [18]. Midline body structures are involved in some patients, resulting from a defective homeotic gene [4,21]. No racial, sexual, or lateral predilection has been known [18]. Ocular and systemic disorders accompanying PA are presented in Table 1 [10,17,18,19] (Figure 2 and Figure 3).

## 4. Examination and Diagnosis

Systemic abnormalities are frequently detected when children are referred to pediatrician after ophthalmic examination and diagnosis of PA. Neurological examination and neuroimaging may be required. If genetic translocations or deletions do exist, or a recessive or dominant pattern of PA or Peters’ plus syndrome is found, parents should consider genetic counseling. Teratogenes such as ethanol and isotretinoin may produce keratolenticular adhesions which clinically correspond to PA [22,23]. Corneal opacification is a feature of congenital rubella syndrome; serological tests for TORCH embryopathy are therefore performed [18].

Complex ophthalmic assessment should include external examination, measurement of intraocular pressure, slit lamp examination, axial length measurement and posterior segment examination by A-scan and B-scan ultrasonography, ultrasound biomicroscopy (UBM), gonioscopy, and indirect ophthalmoscopy. Particular steps may need to be performed in sedation or general anesthesia of the infant. Other than the non-cooperation of the young patient, severe corneal opacity makes the anterior chamber invisible and slit lamp examination can become very difficult. In such cases, anterior segment optical coherence tomography (AS-OCT) and ultrasound biomicroscopy (UBM) become crucial to the confirmation of the diagnosis and the performance of a preoperative assessment of the structures within the anterior chamber, chamber angle, corneal thickness, adhesion, aniridia, or aphakia measurement [24,25]. In posterior segment pathology, electrophysiological tests such as visual evoked responses/potentials (VER or VEP) and electroretinography (ERG) help determine the optic nerve or retinal impairment and, subsequently, the limitations of the considered surgical procedures.

## 5. Treatment

### 5.1. Penetrating Keratoplasty (PK)

**Figure 4:**
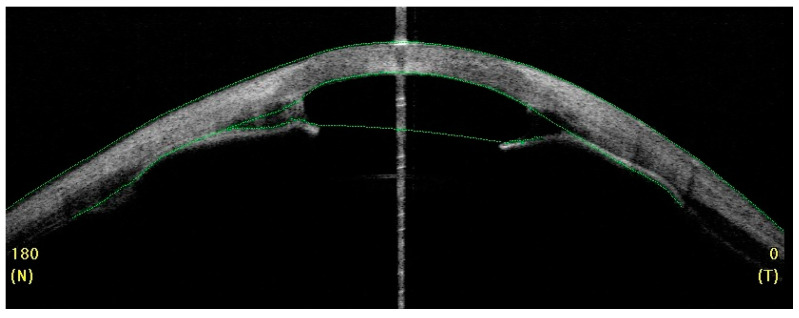
Bilateral PA t.2. LE and OCT anterior chamber 42 months after PK with transparent graft and anterior synechiae.

**Figure 5:**
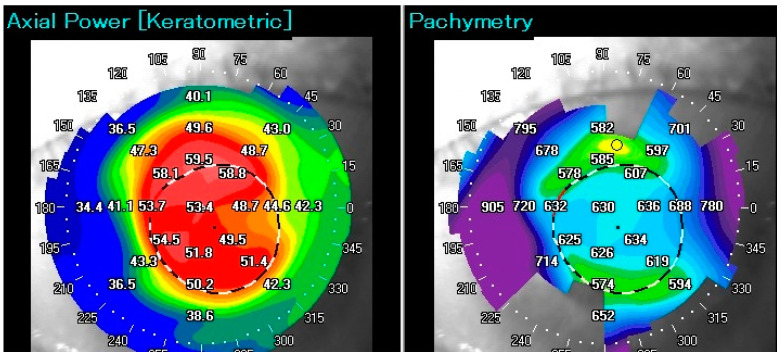
The same patient; postoperative keratometric and pachymetric irregularities. The process of healing in children is rapid and chaotic, resulting in significant postoperative refractive errors. The patient achieved functional vision in LE.

The extent of ocular and systemic involvement, contributing to the general and visual development and rehabilitation of the child, determines the treatment. The ophthalmic management of PA depends on the severity of the opacity. In corneal opacification of a degree that obstructs the light passage and forbids normal visual development in one or both eyes, penetrating keratoplasty, although performed infrequently before the mid-1970s and recommended in pediatric patients with bilateral corneal involvement, is the method of first choice nowadays. The eyes of infants and small children are small, the palpebral fissure is narrow, and the anterior chamber is shallow, and thus, access and manipulation within the surgical field are limited, which makes pediatric keratoplasty very specific in the preoperative, intraoperative, and postoperative periods. Intraocular pressure elevation is the concern during surgery as well as afterwards. Postoperative treatment involves topical corticosteroid and/or cyclosporine A, antibiotics, and lubricants along with systemic cyclosporine A and azathioprine. Examinations under anesthesia (EUAs) in the early postoperative period help to assess the graft status, suture position and tension, intraocular pressure, detection of the inflammation, glaucoma, or retinal detachment and initiate prompt treatment. Typically, the child is examined weekly for the first month and monthly thereafter for the first year. The time of suture removal is variable and determined by the graft status individually. Sutures that loosen in the postoperative period must be removed instantly to prevent infection and vascularization. Infants heal more rapidly than adults, but their inflammatory response also occurs more quickly (Figure 4 and Figure 5). In preverbal children who do not communicate discomfort and vision changes, complications are often discovered at advanced stages [26].

### 5.2. Rehabilitation

Visual acuity is defined as the best visual acuity measured at the last examination. In children with insufficient verbal or cognitive skills, the ability to fixate on and follow visual targets is recorded. Ambulatory vision is defined as vision of counting fingers at a distance of 3 feet or better [8]. The long-term treatment of children with PA is a dual challenge that aims to maintain a clear optical pathway and prevent amblyopia. Although amblyopia does not significantly influence graft survival, it has a major influence on visual outcomes [11,27]. After surgery, parents need to look for any abnormal head posture and eye movements (strabismus, nystagmus). By the age of 7–8 years, called the critical period of neural plasticity, until changes in the lateral geniculate body and the visual cortex are reversible, amblyopia therapy is maximally effective. When the opacity is surgically removed from the optical pathway, the development of central fixation, foveal vision, and functional visual acuity becomes possible. In cases of unilateral disease, after surgery, amblyopia treatment aims to provide a competitive advantage to the operated eye in order to eliminate the inhibitory influence of the better (healthy) eye on the receptive fields of the worse (impaired) one. Occlusion of the better eye, either with a patch or with a cycloplegic atropine, should be started as soon as possible. In marked visual asymmetry between the eyes, or when there is limited visual potential of one eye, patching is more effective than atropine. A patch applied over the skin is preferred to a patch over the spectacles as the child can easily take off the spectacles or look outside through the sides of the occluded eyeglass lens. The incidence of strabismus in bilateral PA was found in 72% cases. It has also been noted that eyes with mild PA respond to amblyopia therapy successfully [16,27,28]. Spectacles or contact lenses are used for the correction of anisometropia and residual astigmatism. Cataract removal is performed as soon as possible. Although some surgeons prefer implanting the intraocular lens at the time of cataract removal, usually a secondary implantation is carried out at the age of 2–3 years of the child. A contact lens is required for temporary aphakia. Optical correction is difficult in infants as the nasal bridge is not fully developed. Adapting the child to spectacles through play is common. Fitting and changing of the contact lens must often be performed in sedation.

### 5.3. Timing of Surgery

The timing for keratoplasty is not precise [10]. Early grafting is performed to prevent amblyopia. Simultaneously, delaying the operation increases the margin of safety associated with general anesthesia, simplifying the surgery and postoperative treatment. Previous studies suggest mixed results for graft survival related to patients’ age at transplantation. One study concluded that poor graft survival could correlate with those younger than 6 months [20]. Others found no significant difference among patients younger than 6 months, aged 6 months to 5 years, and older than 5 years [8]. Another non-randomized study of keratoplasty in patients aged between 1 week and 11.4 years found no benefit of early intervention [29]. The decision to perform surgery at an earlier age depends on the laterality of the corneal opacification and its severity in comparison to the associated ocular or systemic abnormalities and the risk of general anesthesia for the initial surgery and for repeated postoperative EUAs. Family consciousness and their eagerness for long-term cooperation, follow-up visits, occlusion therapy, administration of frequent topical medications, and spectacles or contact lens attendance should contribute to deciding whether and when the surgery may be performed.

## 6. Results

**Figure 6:**
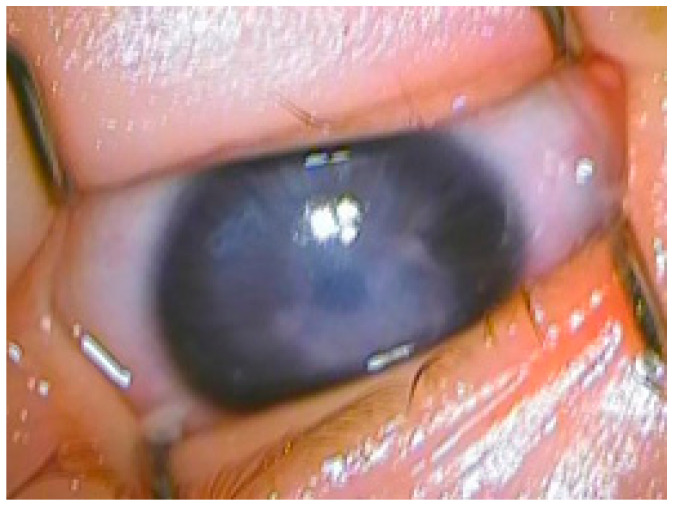
5-week-old infant, LE with suspicion of Peters’ anomaly: ring/doughnut-shaped opacity of central cornea, synechiae between the iris and the posterior border of corneal leukoma.

**Figure 7:**
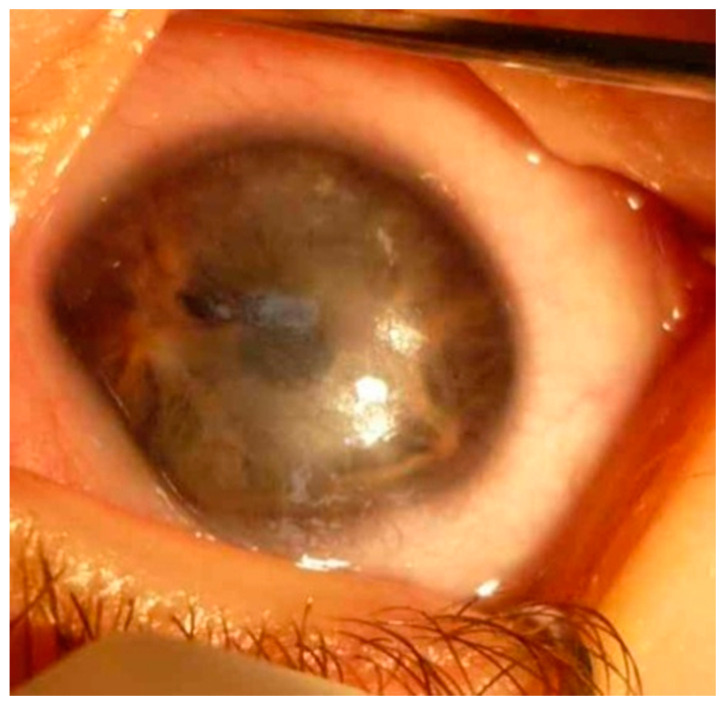
The same child at the age of 13 months. PA type 1. LE: central stromal leukoma with Descemet membrane thickening and fibrosis of 6 mm diameter. Numerous anterior adhesions from the edge of the pupil and the rest of the iris. Peripherally without adhesions. Lens difficult to examine.

**Figure 8:**
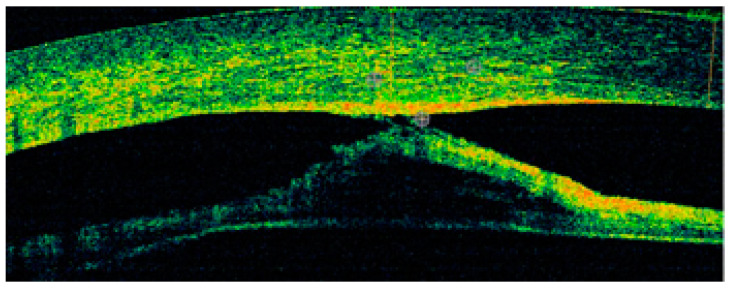
The same patient, LE. OCT: variable corneal thickness, increased within the adhesions (600–700 um), anterior adhesions, corresponding clouding, and defects in the posterior layers of central and paracentral cornea.

The results of keratoplasty in patients with PA are difficult to compare because of the lack of homogenous indications for surgery, involved conditions, size of the study group, and follow-up periods. An attempt to find the graft survival rate and most common predicting factors has been made. Graft survival after penetrating keratoplasty for PA varies widely from 35% to 90%. Among the analyzed studies, one showed that the overall success rate of PK in PA was 43%; the probability of maintaining a clear graft reached 22% at 24 months and remained constant thereafter [20]. Another report recorded a survival rate of 67% at 1 year [8]. A 50% rate of graft failure has also been found [11]. PA type 1, the mild form of the disease, had the best prognosis for graft clarity (Figure 6, Figure 7 and Figure 8).

A report suggested that out of 30 eyes operated with PA type 1, 27 (90%) eyes (including re-grafts) maintained clear grafts [16]. According to the authors, the best results can be achieved if the surgery is performed between 2 and 12 months of age; however, preoperative patient selection according to disease severity is crucial for this outcome [16]. Bhandari et al. [3] reported an overall success rate of 53% in 15 eyes, with a significantly higher success rate in PA type 1 (87.5%) than type 2 (14.2%), which is in accordance with Zaidman et al. [16]. Another retrospective study of 14 children who underwent keratoplasty for unilateral PA showed overall graft clarity in 11 eyes (78.6%) [30]. Chang et al. revealed that among 22 patients, graft failure rates at 1 year, 3 years, 5 years, and 10 years after PK were 30%, 39%, 70%, and 77%, respectively [19]. Successful corneal transplantation was found to be measured not only by the parameter of graft clarity, but also by postoperative visual acuity. Approximately 50% of infants and small children who undergo PK for congenital corneal opacities achieve functional vision postoperatively, which is defined as the ability to count fingers, navigate, eat independently, and recognize persons [31]. Final best-corrected visual acuity (VA) measured with Snellen charts in children with bilateral disease ranged from 20/25 to no light perception (NLP). It was better than 20/400 in 37% of operated eyes, but 19% developed NLP [23]. One report found that 78% of grafted eyes presented with VA 20/200 or less at the time of the most recent follow-up [11]. In another study, 10% had VA 20/100 or better. These children had mild or moderate disease and had received only one graft, which was clear at the time of final follow-up. VA 20/200 to 20/400 was found in 19% of cases and 71% showed CF or worse. One eye with best VA of 20/25 was found after surgery in unilateral disease, whereas all eyes with LP or NLP were mainly found after bilateral surgery. At least HM visual acuity was achieved in all eyes with a clear graft [29]. Interestingly, according to Zaidman et al., in patients older than 3 years of age with an ability to communicate, more than 50% had VA 20/100 or better, and two-thirds of patients had VA 20/400 or better [16]. No patient had VA worse than HM, and there were no cases of phthisis or NLP. Presumably, again, preoperative patient selection was crucial for the outcome and the rate of severe PA type 2 patients contributed to the poorer results. Children with PA and concomitant ocular disorders requiring additional surgical procedures such as anterior segment reconstruction, lensectomy, or vitrectomy have worse outcomes of PK [11,20,29,32].

## 7. Reasons of Graft Failure

The main reason for graft failure in PA is rejection (65%) [11,20]. Graft rejection has been observed to be reversible in only 27–28% of episodes [20,29], compared with a rate of 50–78% in adults. Pediatric patients have an increased irreversible rejection rate because of their highly active immune systems. Over 90% of rejection episodes occur within the first year [20]. Parents must therefore be trained to monitor the child’s eye daily for conjunctival congestion, haziness, adherence of mucus to the graft, or any unwillingness of the child to open the eye. The incidence of graft infection varies from 10 to 50% in pediatric grafts. If the infected graft survives, prognosis for VA is no better than HM [33]. Most infections are caused by loose sutures and irritation. Non-compliance to follow-up and delays in reporting to the ophthalmologist after the onset of symptoms are the most important risk factors for graft infection and rejection [20]. Congenital and secondary glaucoma have been shown to be an independent predictor of graft failure [8,16]. Increased intraocular pressure is related to endothelial cell damage and affects graft survival. Retinal detachments and phthisis resulting in NLP in eyes that underwent trabeculectomy for glaucoma management have been reported [19]. Complications such as secondary glaucoma, epithelial defects, band keratopathy, retinal detachment, wound leakage, retrocorneal membrane, or microbial keratitis are some of the reasons that cause graft rejection and therefore induce the necessity of re-grafting, although it has been noted that the survival rate of a re-graft is low. Rejection reversals tend to be even less successful in re-grafts than in primary grafts [11]. Repeat grafts may still be indicated in children in the amblyogenic age to temporarily enhance their visual development. The results of corneal grafting in PA prompt some surgeons to exclude patients with monocular disease from PK. However, an eye with even slight vision is crucial in case of pathology or trauma of the fellow eye [30].

## 8. Alternatives to Penetrating Keratoplasty

Posterior lamellar keratoplasty/endothelial keratoplasty, like Descemet stripping automated endothelial keratoplasty (DSAEK), may be performed in appropriately selected cases of PA [34]. To restore normal endothelial function, make the posterior corneal surface more regular and smoother, and thus achieve regress in corneal edema and clouding density, a fully functional and healthy posterior layer of donor tissue is transplanted. DSAEK in such cases has advantages that include undisturbed anterior layers, no corneal sutures, reduced postoperative astigmatism, and visual recovery within 6–12 weeks, which is achieved between 6 and 12 months after PK. However, identified limitations include stromal opacity that cannot be healed; manipulation inside the small AC which may cause lens damage; and postoperative positioning requiring the patient to lie flat for a few days until the air bubble present in the AC allows the graft to heal properly. Further studies on DSAEK in PA are needed.

### Partial Corneal Opacity or Disqualification from PK

**Figure 9:**
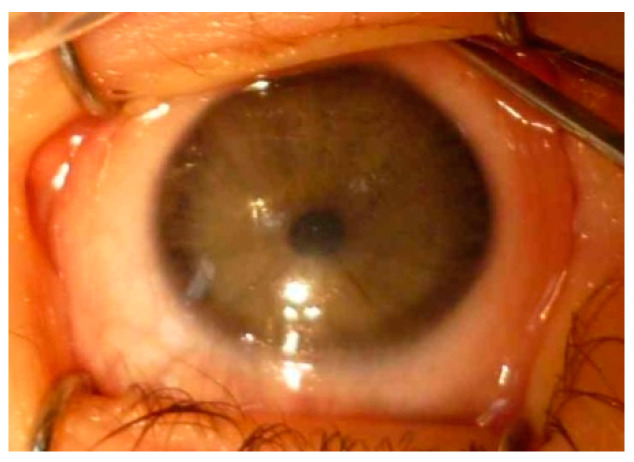
The patient, RE: discreet corneal clouding in visual axis, thready irido-corneal adhesion at 5 o’clock, gentle deformation of the pupil—upwards decentration, correct mydriasis, no lens pathology.

Optical sector iridectomy, pupil dilation, or corneal autorotation aim at providing ambulatory vision and reduce the likelihood of amblyopia for patients awaiting or disqualified from PK. The advantages observed with optical iridectomy are that it avoids sutures, graft rejection, or postoperative glaucoma, provided there are no cataracts and there is a suitable area of maximum clear peripheral cornea selected for the iridectomy site. Superior quadrants are least preferred since they are hidden by the lids [35,36,37,38]. In our clinic, optical sector iridectomy was performed in two children with bilateral PA and both patients achieved functional vision without PK (unpublished data). Pupil dilation with corneal rotation may be considered in the management of full-thickness partial opacifications. Corneal autorotation after pupillary enlargement removes the opacity from the visual axis and relocates it to the superior quadrant [39] (Figure 9).

## 9. Pediatric Keratoprosthesis 

Satisfying results of pediatric keratoprosthesis in PA have been described [40,41,42]. In the past, keratoprosthesis implantation was an infrequent procedure used as a last rescue, but technical modifications of the device as well as progress in surgical and postoperative care have made it more successful. The remarkable retention rate and low incidence of postoperative complications reported by Aquavella et al. are promising [41]. Keratoprosthesis is a viable means of managing repeat graft failure. A clear optical pathway and stable refractive error can be obtained from the first days. The device, however, when mounted on donor tissue, requires life-long care. Topical medications and a bandage contact lens protect it from infection and are necessary to obtain a quiet optical surface. There is no risk of secondary cataract as the biological lens is removed during the operation. The main complication is retroprosthetic membrane formation, treated either with laser or surgery. Patients are strictly monitored for glaucoma. Furthermore, vitreo-retinal and glaucoma operations are frequently needed.

## 10. Summary

PA is rare, but an accurate ophthalmologic diagnosis is vital to predict its natural history, find associated abnormalities, provide genetic counseling, and decide on the appropriate medical or surgical therapy. All patients with PA should be viewed for systemic malformations, especially if other ocular defects are present or if the finding is bilateral. Penetrating keratoplasty remains the surgery of choice for the management of PA. Visual acuity is eventually influenced by accompanying ocular and systemic disorders, including those related to intellectual development, which contribute to overall difficulties in childcare. Patients with PA call for an integrated team approach, which includes corneal surgeons, pediatric ophthalmologists, anesthesiologists, and pediatricians. Surgical intervention should be attempted when parents fully understand the limited visual potential and necessity of long-term follow up.

## 11. Methods of Literature Research

This study included an electronical search of the PubMed and Embase databases with the key words “Peters’ anomaly”, “Peters anomaly” published between the years 2000 and 2022. The selected records were articles characterizing the disease and treatment as well as results of treatment and follow-up in small groups of patients, which were studied and compared. Searching with the key words “pediatric keratoplasty” and “pediatric keratoprosthesis” found a few reviews and another series of patients. Information concerning patients with Peters’ anomaly was extracted. Articles following a supplementary search that did not provide sufficient information or data pertaining to this article were excluded. 

## Figures and Tables

**Table 1:** Ophthalmic and systemic abnormalities presented in patients with Peters’ Anomaly.

OPHTHALMIC	microphthalmia, iridocorneal adhesion, corectopia, keratolenticular touch, anterior polar cataract, aniridia, aphakia, iris hypoplasia, iris coloboma, chorioretinal coloboma, staphyloma, retinal dysplasia, ptosis, persistent hyperplasic primary vitreous, optic nerve hypoplasia, foveal hypoplasia, macular pigment epitheliopathy, sclerocornea
SYSTEMIC	developmental delay, central nervous system defects, craniofacial abnormalities, microcephaly, hypopituitarism, dwarfism, cardiac malformations, skeletal deformities, genitourinary malformations, ear defects, cleft lip and palate, fetal alcohol syndrome, autism
